# Performance Evaluation of Recycled Aged Rubber Modified Asphalt Mixtures with Soybean Oil Rejuvenator

**DOI:** 10.3390/ma19091893

**Published:** 2026-05-04

**Authors:** Kwadwo Ampadu Boateng, Meng Wu, Dongzhao Jin, Dayo Sunkami Olatunde, Zhanping You

**Affiliations:** Department of Civil, Environmental, and Geospatial Engineering, Michigan Technological University, 1400 Townsend Drive, Houghton, MI 49931-1295, USA; kwadwob@mtu.edu (K.A.B.); mewu@mtu.edu (M.W.); dongj@mtu.edu (D.J.); dsolatun@mtu.edu (D.S.O.)

**Keywords:** rubber-modified asphalt, cracking resistance, rutting performance, rejuvenation, soybean oil rejuvenator

## Abstract

**Highlights:**

- Soybean oil restores cracking resistance in aged rubber asphalt.
- Rejuvenation reduces rutting resistance due to softening.
- Space diagrams show that balancing aging, modification, and rejuvenation improves mix performance.
- Dosage must be optimized to avoid rutting failures.
- Rejuvenated aged rubber asphalt is viable for recycled mix design.

**Abstract:**

This study evaluates the performance of recycled, long-term-aged rubber-modified asphalt (RMA) mixtures rejuvenated with soybean oil. Crumb rubber is widely used in asphalt mixtures for its ability to enhance elasticity, crack resistance, and durability. However, long-term aging leads to oxidative hardening, increased stiffness, and reduced cracking resistance, creating a need for effective rejuvenation strategies. To simulate extended field aging, plant-produced RMA mixtures were conditioned at 85 °C for five and ten days and subsequently treated with 10% soybean oil by binder weight. Mechanical performance was assessed using the Disc-Shaped Compact Tension test, Indirect Tensile Asphalt Cracking Test, Hamburg Wheel Tracking Test, and Rapid Shear Rutting Test. Rejuvenation effectively reversed aging-related deterioration, increasing fracture energy by 137–211% and improving cracking tolerance indices by 22–104%, thereby restoring or surpassing the cracking performance of unaged RMA mixtures. This improvement in flexibility was accompanied by reduced rutting resistance, with rutting tolerance indices decreasing by 52–70%, consistent with the softening effect of bio-based oils. Performance space diagrams further illustrated the trade-off between enhanced cracking resistance and increased rut susceptibility. Overall, the results demonstrate that soybean oil provides strong restorative capabilities for aged RMA mixtures, but achieving balanced field performance requires optimization of rejuvenator dosage.

## 1. Introduction

The use of rubber-modified asphalt (RMA) addresses critical challenges in pavement construction by enhancing both environmental sustainability and pavement performance. Incorporating recycled rubber, typically sourced from scrap tires, into asphalt mixtures reduces landfill waste and minimizes the environmental impact associated with tire disposal. This eco-friendly approach supports circular economy principles by repurposing waste materials. From a performance perspective, RMA improves the elasticity and flexibility of pavements, increasing resistance to cracking, rutting, and deformation under heavy traffic loads and temperature fluctuations [1]. These enhancements extend the service life of road surfaces, reduce maintenance frequency, and lower lifecycle costs. Consequently, RMA offers a sustainable and durable solution that aligns infrastructure development with environmental conservation goals.

Performance-wise, RMA improves multiple critical pavement properties. Incorporation of crumb rubber at optimized dosages, such as around 10–15%, has demonstrated enhancements in rutting resistance by up to 25%, fatigue resistance by 30%, and rheological stability by 15% compared to conventional asphalt binders [2,3]. These modifications result in pavements that are more durable and better able to withstand deformation and cracking under heavy traffic and harsh environmental conditions. Further, RMA mixtures exhibit greater binder stiffness and viscosity, contributing to improved resistance to permanent deformation, such as rutting, at elevated temperatures [4]. This means RMA pavements can maintain structural integrity over longer service periods with reduced maintenance needs. The fatigue life of pavements is also extended due to the elastomeric properties of rubber, which absorb and dissipate stresses more effectively than traditional binders [3]. From an environmental perspective, the use of crumb rubber in asphalt offers a valuable recycling pathway for waste tires, reducing landfill disposal and mitigating environmental hazards associated with tire waste [5,6,7,8]. RMA pavements align with sustainable construction practices by lowering the carbon footprint of pavement materials and enabling the reuse of industrial waste [2,5]. Additionally, these pavements exhibit reduced sensitivity to temperature, implying better performance stability under climate variations, which is increasingly important under changing climate conditions [9].

The growing need for sustainable and economical pavement materials has stimulated extensive research into the reuse of aged RMA mixtures. Recycled RMA mixtures produced from waste tires provide notable environmental advantages by reducing landfill disposal and conserving natural resources. Additionally, the recycling of asphalt mixtures reduces CO_2_ emissions associated with the use of new materials, thereby contributing to the attainment of climate neutrality [10,11,12].

Nevertheless, the inclusion of aged RMA mixtures often introduces challenges such as increased stiffness and brittleness, which can negatively affect long-term pavement performance. To mitigate these issues, rejuvenating agents have been employed to restore the properties of aged binders and improve mixture durability. Among the available options, soybean-based rejuvenators have attracted significant interest due to their environmentally friendly nature and their ability to enhance the rheological properties of aged asphalt binders.

Rejuvenators and rejuvenating agents restore the performance of hot-mix asphalt (HMA) primarily by reversing the detrimental effects of aging in reclaimed asphalt pavement (RAP). Aging in asphalt binders occurs due to oxidation and the loss of volatile components during service life, resulting in stiffening, embrittlement, and reduced flexibility, which lead to cracking and reduced durability of HMA with high RAP content [13,14]. The rejuvenating agents work by physically and chemically interacting with the aged binder to restore its maltene fractions and reduce asphaltene content, effectively replenishing the binder’s original composition. This increases the flexibility and penetration of the binder while lowering viscosity, thereby improving the low-temperature cracking resistance and overall deformation performance of the asphalt [14,15]. Many rejuvenators act physically rather than chemically, mixing with the aged binder to soften it and restore its micro-morphology, as confirmed by microscopic and spectroscopic analyses, such as Fourier-transform infrared spectroscopy (FTIR) [15].

In practical terms, incorporation of bio-based or oil-based rejuvenators (e.g., pongamia oil, composite castor oil, vegetable oils) into aged asphalt binders decreases complex shear modulus, which in turn improves rheological properties, including ductility and fatigue resistance. This treatment results in rheological responses similar to, or sometimes better than, those of virgin binders, as rejuvenators reduce binder stiffness and increase toughness, thereby better withstanding traffic loading and climatic stresses [16,17]. On the mixture scale, rejuvenators help high RAP content mixes (50% or more RAP) achieve performance comparable to conventional low RAP mixes by reducing modulus, moisture susceptibility, tensile strength, and cracking resistance. Laboratory tests, including complex modulus measurements and semicircular bending tests, support that rejuvenators can effectively restore the mechanical performance and durability of recycled HMA with significant RAP fractions [13,18]. The rejuvenation process also involves optimizing the type, dosage, and blending methods of rejuvenators, as these factors influence the final binder and mixture behavior. Chemical characterization techniques help ensure that the rejuvenator has adequately restored the aged binder’s properties and can withstand further aging.

While the rejuvenation of aged conventional asphalt mixtures has been widely studied, comparatively little attention has been given to the treatment of aged RMA mixtures. This study aims to address this gap by systematically evaluating the performance of recycled, long-term-aged RMA mixtures rejuvenated using soybean oil. The mechanical and durability characteristics of these mixtures were examined to determine the feasibility of combining recycled RMA mixtures with a renewable rejuvenating agent to produce sustainable, high-performance pavement materials. The findings of this research are intended to support the advancement of innovative asphalt mixture designs that simultaneously address environmental sustainability and long-term pavement performance.

To achieve these objectives, both a conventional control mixture and an RMA mixture were investigated to evaluate the effects of extended aging and the effectiveness of soybean oil as a rejuvenating agent. Plant-produced mixtures were compacted in the laboratory using a Superpave gyratory compactor (SGC) and subsequently conditioned in a forced-draft oven at 85 °C for periods of 5 and 10 days to simulate long-term aging (LTA). After aging, rejuvenators were incorporated into the mixtures to assess their ability to restore performance. Rutting resistance and moisture damage potential were evaluated using the Hamburg Wheel Tracking Test (HWTT), while low-temperature cracking resistance was measured with the Disc-Shaped Compact Tension (DCT) test. Additional characterization of cracking and rutting behavior was conducted using the Indirect Tensile Asphalt Cracking Test (IDEAL-CT) and the Rapid Shear Rutting Test (IDEAL-RT), respectively.

## 2. Materials and Methods

### 2.1. Materials

For this study, all binder and loose asphalt mixtures were obtained from an asphalt plant located at 1503 Pine St., Essexville, MI 48732, USA. These materials were transported to the laboratory, where they underwent controlled LTA and were subsequently treated with soybean oil as a rejuvenating agent. Two distinct mixture types were examined to evaluate the effects of aging and rejuvenation across different paving systems. These included a conventional asphalt mixture, referred to as the Control mix, and designed to satisfy the Michigan Department of Transportation (MDOT) 4EML requirements [19], and an RMA mixture, referred to as the Rubber mix and designed in accordance with MDOT 5EML specifications [19].

The 4EML mixture incorporates a nominal maximum aggregate size (NMAS) of 12.5 mm and is commonly used as a surface or leveling layer. It is typically placed at application rates ranging from 119 to 149 kg/m^2^ (220–275 lbs/yd^2^) and is intended for pavements experiencing low to moderate traffic levels, corresponding to approximately 0.3–3.0 million equivalent single-axle loads (ESALs). By comparison, the 5EML mixture features a smaller NMAS of 9.5 mm and is primarily used as a surface course. This mixture is applied at lower rates of 90–119 kg/m^2^ (165–220 lbs/yd^2^) but is designed for the same ESAL range, making it suitable for evaluating the influence of rubber modification under comparable traffic conditions.

To establish reference performance, the control mixture was produced using a performance grade (PG) 64-28 asphalt binder at a total binder percentage of 5.5% and mixed at a temperature of 155 °C. The corresponding aggregate gradation is presented in Table 1. This mixture provides a baseline for assessing the effects of LTA and rejuvenation relative to the RMA system.

**Figure 1:**
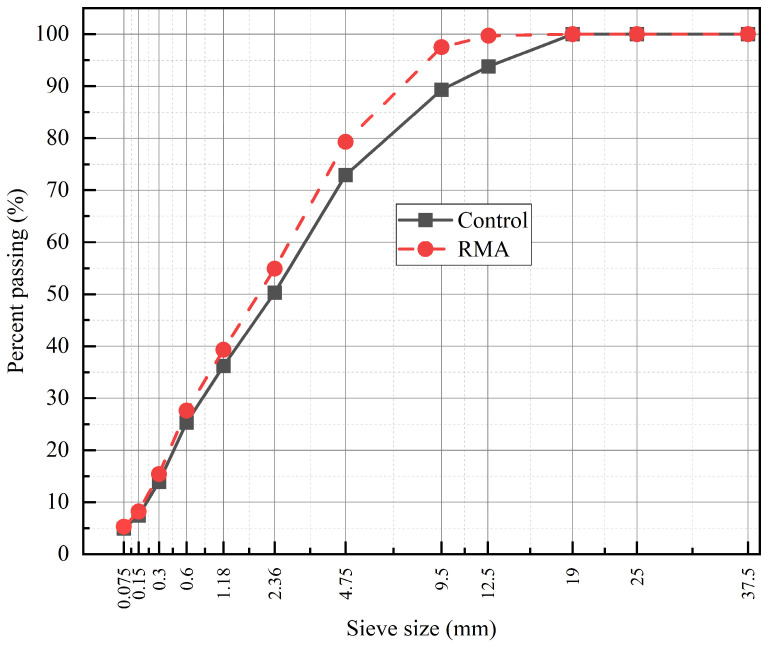
Particle size distribution curve of the Control and RMA mixture.

The rubber-modified mixture was manufactured using a PG 52-28 binder produced through the wet-process rubber modification method. The modified binder consisted of 22% crumb rubber blended with 78% base binder by weight, resulting in improved elastic behavior and altered rheological characteristics. The total binder content for the 5EML RMA mixture was 5.6%, and a higher mixing temperature of 175 °C was employed to promote effective rubber–binder interaction and uniform dispersion. The aggregate gradation for the RMA mixture is provided in Table 2. Differences in binder composition and aggregate structure between the two mixtures facilitate a comprehensive assessment of the combined effects of rubber modification, aging, and rejuvenation on mixture mechanical and durability performance. The aggregate particle-size distribution curves for both mixtures are shown in Figure 1.

### 2.2. Sample Preparation

The conventional MDOT control mixture and the RMA mixture were collected from the construction site and delivered to the laboratory for testing. Sample preparation followed the Superpave volumetric mix design protocol to ensure uniform compaction and consistent material properties across all specimens. For all performance tests, the target air-void content was maintained at 7% with an allowable tolerance of ±0.5%, thereby minimizing variability among testing conditions. Test specimens intended for HWTT, IDEAL-RT, IDEAL-CT, and DCT tests were compacted using SGC. Each specimen was fabricated to a nominal diameter of 150 mm, with heights of 60 mm for HWTT, 62 mm for both IDEAL-RT and IDEAL-CT, and 50 mm for DCT specimens. The required mass for each specimen was determined through back-calculation using the theoretical maximum specific gravity (Gmm). Target specimen mass was computed as 93% of Gmm multiplied by the specimen volume and subsequently adjusted by applying a correction factor, generally ranging from 0.965 to 0.985, to compensate for surface void effects. The selected correction factor varied based on specimen geometry and aggregate gradation, ensuring that laboratory-compacted specimens accurately reflected field compaction conditions.

#### 2.2.1. Aging Protocol

To replicate the aging mechanisms that asphalt mixtures undergo in the field, standardized laboratory conditioning procedures were applied [20,21]. The American Association of State Highway and Transportation Officials (AASHTO) standard specification, AASHTO R30-15 protocol, is widely used to simulate both short-term aging (STA) and LTA [21] and was adopted in this study alongside the more recent AASHTO R121-24 standard for long-term laboratory conditioning of asphalt mixtures [22]. Under R 30-15, STA is achieved by heating loose asphalt mixtures in a forced-draft oven at 135 °C for four hours, capturing the volatilization and oxidation that occur during production and placement. LTA is then simulated by conditioning the short-term-aged mixture at 85 °C for 5 days, representing approximately 5–10 years of field aging. In this work, Method B of AASHTO R 121-24 and the procedures outlined in AASHTO R30-15 were used to age both the conventional control mixture and the RMA mixtures. Conditioning durations of 5 and 10 days were selected to represent roughly 5–10 years and 10–20 years of field performance, respectively.

#### 2.2.2. Recycling and Rejuvenation Protocol

The mixture samples, conditioned at 85 °C for 5 or 10 days, were removed from the oven upon completion of the aging protocol. Before specimen fabrication, the aged mixtures were reheated to 155 °C to restore adequate workability. 100% pure soybean cooking oil supplied by a food services company in Zeeland, Michigan, was used as a rejuvenator.

**Figure 2:**
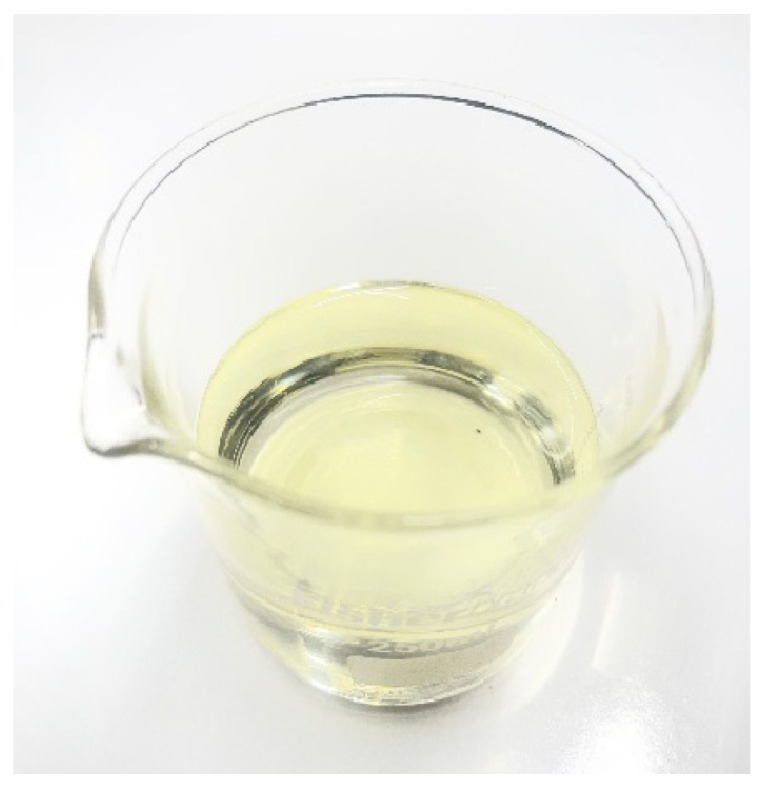
Soybean cooking oil used as a rejuvenator.

The soybean cooking oil shown in Figure 2 was used as a rejuvenator and incorporated at a dosage of 10% by weight of the binder to counteract stiffness induced by long-term oxidative aging and enhance mixture performance. 10% dosage of bio-rejuvenator has been found to reduce oxygenated-group intensity and reduce asphaltene content, restoring the binder to a more “original” state [23,24]. The rejuvenator and aged RMA mixtures were thoroughly blended using a bucket mixer to ensure uniform dispersion. Following blending, the rejuvenated mixtures were conditioned in the oven for an additional 2 h and subsequently compacted at 145 °C to prepare specimens for mechanical testing.

### 2.3. Mixture Performance Evaluation

#### 2.3.1. Hamburg Wheel Tracking Test

The rutting resistance and moisture susceptibility of the asphalt mixtures were assessed using the HWTT in accordance with AASHTO T 324-14 [25]. Cylindrical specimens with a nominal diameter of 150 mm and a height of approximately 62 mm were prepared using SGC, with four parallel specimens tested for each mixture and condition. The HWTT was performed using a rolling steel wheel applying a repeated load of 703 ± 4.5 N at a frequency of 52 ± 2 passes per minute. During testing, specimens were fully submerged in water maintained at 50 °C to simulate combined moisture damage and high-temperature loading. Rut depth was continuously monitored using a linear variable differential transformer (LVDT). The test was stopped upon reaching either a maximum rut depth of 20 mm or 20,000 wheel passes, whichever occurred first.

#### 2.3.2. Disc-Shaped Compact Tension (DCT) Test

The cracking resistance at low temperatures was evaluated using the DCT test, per the standard specification of the American Society for Testing and Materials (ASTM) D7313-20 [26]. Three replicate cylindrical specimens (150 mm diameter) were compacted using SGC, then cored and sawed to produce DCT samples with a breadth of 50 mm. A central vertical notch was introduced using a diamond saw to ensure controlled initiation of the crack. All specimens were kept at −18 °C prior to testing to achieve thermal equilibrium, consistent with the binder’s low-temperature performance grade plus 10 °C. This temperature was selected to accommodate both the MDOT control mixture (PG 64-28) and the RMA mixture (PG 52-28).

The DCT test was conducted under displacement-controlled conditions using a constant loading rate, while load response and crack mouth opening displacement (CMOD) were continuously measured throughout the test. Loading was continued until full specimen failure occurred. The fracture energy (Gf) was determined by integrating the load–displacement response and normalizing the resulting energy by the ligament area. Fracture energy was adopted as the primary indicator of low-temperature cracking performance, with higher values reflecting increased resistance to crack initiation and propagation.

#### 2.3.3. Indirect Tensile Asphalt Cracking Test (IDEAL-CT) Test

The cracking behavior of the asphalt mixtures was characterized using the IDEAL-CT in accordance with ASTM D8225-19. Test specimens were produced using SGC, resulting in cylindrical samples with a nominal diameter of 150 mm and a height of approximately 62 mm. Three replicate specimens were prepared for each mixture. Before testing, specimens were equilibrated at 25 °C to ensure consistent thermal conditions.

The IDEAL-CT was performed under displacement-controlled loading at a fixed rate of 50 mm/min. Compressive loading was applied along the vertical diametral plane of the specimen, generating tensile stresses that led to crack development and eventual failure. Throughout the test, force and deformation responses were continuously recorded. Cracking resistance was quantified using the Cracking Tolerance Index (CT Index), which is calculated from the post-peak portion of the load–displacement curve. Higher CT Index values correspond to greater resistance to crack initiation and propagation.

#### 2.3.4. Rapid Shear Rutting Test (IDEAL-RT)

Rutting performance of the asphalt mixtures was characterized using the IDEAL-RT following ASTM D8360-22 [27]. Cylindrical specimens measuring 150 mm in diameter and approximately 62 mm in height were fabricated using SGC, with three replicates prepared for each mixture. Before testing, specimens were stored at 50 °C to ensure uniform temperature conditions.

Testing was carried out under displacement-controlled conditions, during which a cyclic vertical compressive load was applied across the specimen diameter to induce shear deformation and permanent strain accumulation. Force and displacement responses were continuously monitored throughout the loading process. Rutting resistance was evaluated using the Rutting Tolerance Index (RT Index), calculated from the load–displacement behavior. Higher RT Index values indicate greater resistance to permanent deformation.

## 3. Results and Discussion

### 3.1. Cracking Performance

#### 3.1.1. Disc-Shaped Compact Tension (DCT) Results

**Figure 3:**
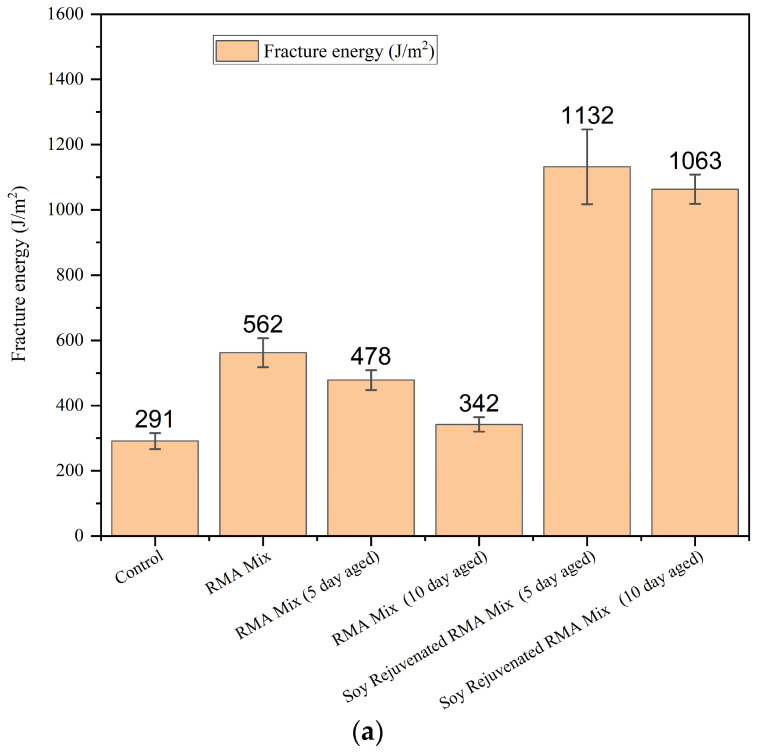

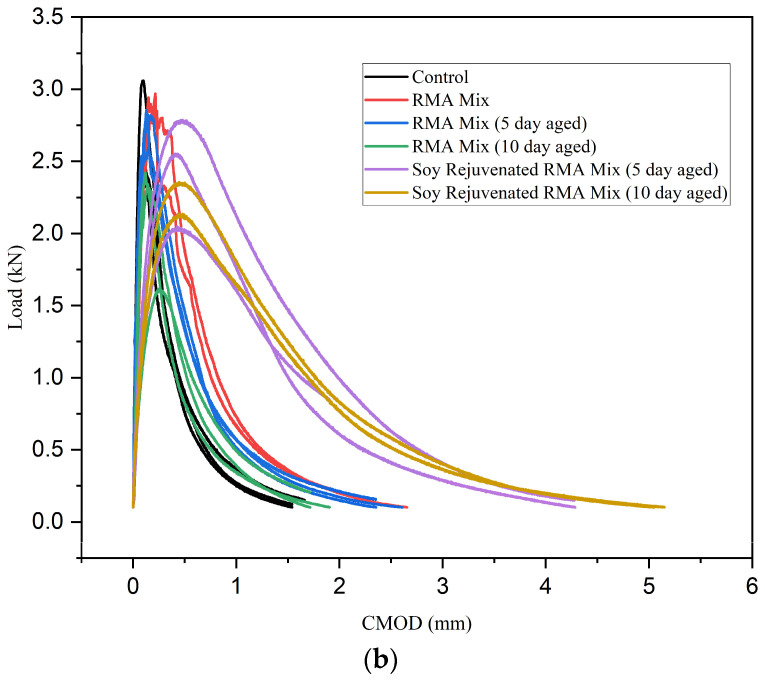
DCT test results, (**a**) fracture energy of DCT samples at −18 °C, (**b**) load vs. CMOD.

Figure 3 presents the fracture energy values for the Control, RMA mix, aged RMA mixes (5-day and 10-day), and the corresponding soybean-rejuvenated mixes. The RMA mix exhibited consistently higher fracture energy than the Control mixture. This is attributed to the wet-process crumb rubber modification, which enhances low-temperature cracking resistance by improving energy dissipation associated with the elasticity of swollen rubber particles [28]. LTA (5 and 10 days at 85 °C) resulted in a clear reduction in fracture energy, demonstrating the typical embrittlement behavior induced by oxidative aging. The reason is that aging promotes molecular stiffening, asphaltene agglomeration, and loss of maltenes, leading to reduced crack tolerance, a behavior commonly observed during the LTA of RMA binders and mixtures [29,30].

Rejuvenation using soybean oil significantly increased the fracture energy of both aged mixtures. Soybean oil rejuvenation produced a substantial recovery in cracking performance for both 5-day and 10-day aged RMA mixtures. DCT fracture energy increased by 137% for the 5-day aged mixture and by 211% for the 10-day aged mixture.

In both cases, the fracture energy exceeded that of the unaged RMA Mix, highlighting the strong rejuvenation efficacy of soybean-derived oils. Previous studies for base asphalt have shown that soybean-based rejuvenators restore binder rheology by replenishing light fractions, improving molecular mobility, and facilitating the dispersion of aged asphaltene clusters through physical interactions and enhanced diffusion [15,31,32]. This phenomenon was also observed in the RMA samples tested. The magnitude of fracture-energy recovery observed here demonstrates that bio-oil rejuvenators are particularly compatible with RMA and aged binders.

#### 3.1.2. Indirect Tensile Asphalt Cracking Test (IDEAL-CT) Results

**Figure 4:**
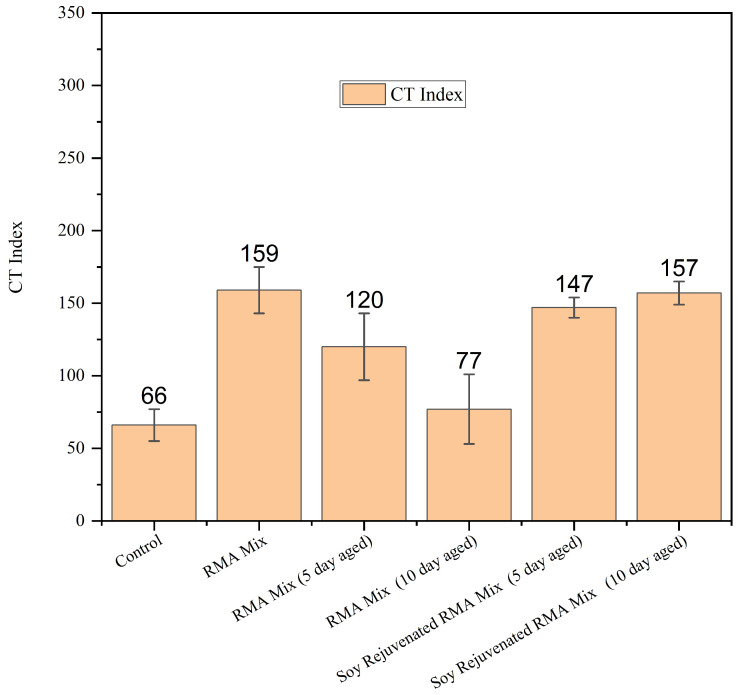
IDEAL-CT results for control, RMA, aged RMA, and soybean rejuvenated RMA mixtures.

Figure 4 presents the IDEAL-CT results for control, the unaged RMA mix, the long-term aged RMA mixes (5 and 10 days aged at 85 °C), and their corresponding soybean oil rejuvenated mixes. The control mixture exhibited the lowest CT index. The unaged rubber mix showed a significant increase in CT index; this is due to the ability of rubber modification to enhance crack resistance, as increased elasticity has been reported in the literature [3,28,33]. LTA led to a drop in the CT Index for the 5-day-aged rubber mixture. The CT Index was further reduced in the 10-day-aged rubber mixture. This is attributed to the loss of saturates and aromatics during oxidative aging, which culminates in increased brittleness and stiffness, leading to crack initiation and propagation [29,30,34]. Soybean oil rejuvenation effectively restored the cracking resistance lost through aging. Both the 5- and 10-day-aged soybean-rejuvenated RMA mixtures had their CT indices improved by 22.5% and 104% for the 5-day and 10-day aged mixtures. These recoveries align with the role of bio-based rejuvenators in replenishing light fractions, reducing effective stiffness, and improving molecular mobility, thereby enhancing tensile strain accommodation and delaying crack propagation [14,15,16,31]. The greater recovery observed in the more severely aged (10-day) mixture suggests a larger “capacity” for softening when the binder network has undergone more extensive oxidation, an effect reported for bio-oil treatments of aged RAP/RMA systems [14,31,32].

### 3.2. Rutting Performance

#### 3.2.1. Hamburg Wheel Tracking Test (HWTT) Results

**Figure 5:**
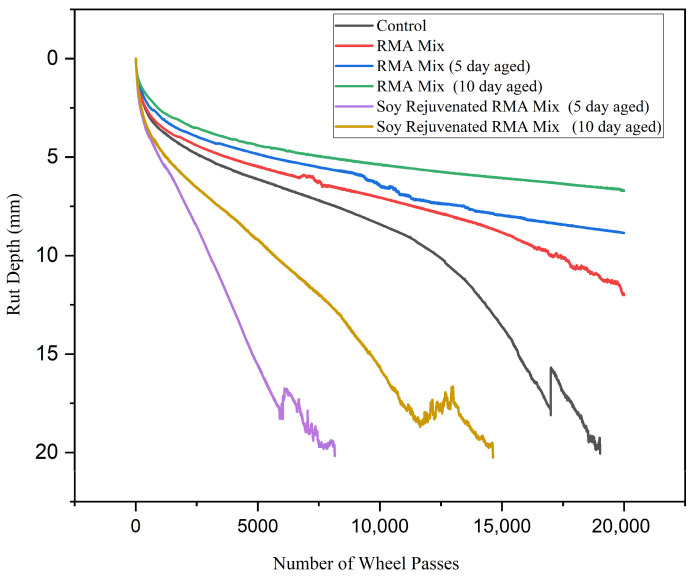
HWTT curves for control, RMA, aged RMA, and soybean rejuvenated RMA mixtures.

Figure 5 presents the rutting performance of Control, RMA mix, aged RMA mixes (5-day and 10-day), and the corresponding soybean-rejuvenated RMA mixes. RMA mixtures typically exhibit better rutting resistance than other mixtures because rubber modification increases binder viscosity, enhances elastic recovery, and improves the shear resistance of asphalt mixtures [2,3,4,5]. Owing to this, the RMA mix exhibited superior rutting resistance compared to the control mixture, with lower rut depths and delayed progression under submerged, high-temperature loading.

Long-term aging substantially increased rutting resistance for the RMA mix. Both 5-day- and 10-day-aged specimens showed shallower rut depths and delayed progression toward the 20 mm failure criterion. These trends are well supported by prior investigations documenting the stiffening and deformation-resistant behavior imparted by oxidative aging in RMA mixtures [29,30,35,36]. The 10-day-aged RMA mix demonstrated the highest rutting resistance among all mixtures. This is because extended oxidative conditioning improves shear stability in RMA [30,34].

In contrast, rejuvenated mixtures exhibited significantly higher rut depths, steeper rutting, and earlier failure. Bio-oil rejuvenators can decrease mixture stiffness, increase susceptibility to plastic flow, and accelerate stripping-related deformation when exposed to water and high temperatures [14,16,17,37]. The HWTT curves observed in this study match those documented in rejuvenator-based evaluations of aged RAP and RMA mixtures, where improved flexibility is achieved at the expense of rutting resistance [13,14,37].

#### 3.2.2. Rapid Shear Rutting Test (IDEAL-RT) Results

**Figure 6:**
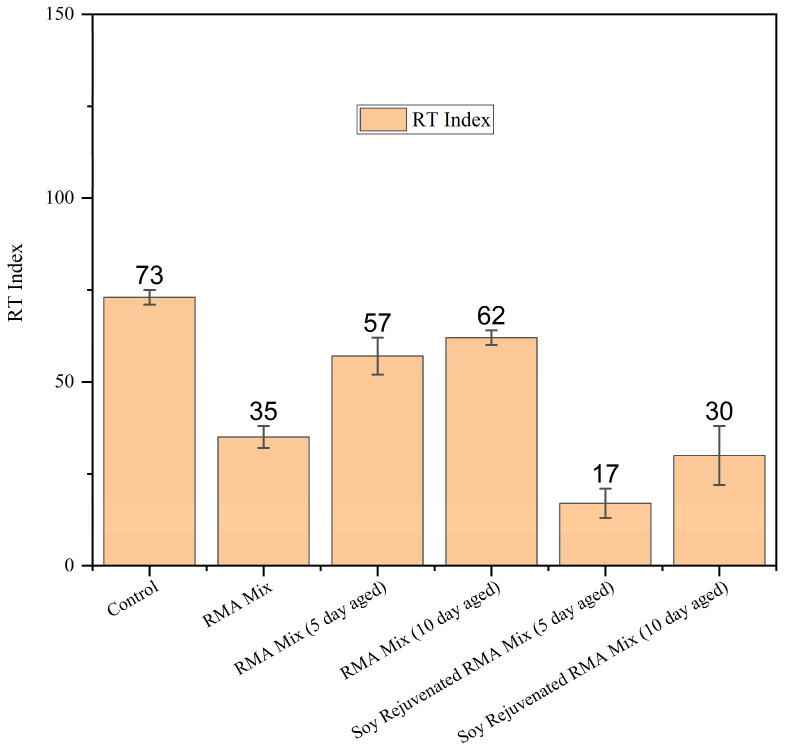
IDEAL-RT results for control, RMA, aged RMA, and soybean rejuvenated RMA mixtures.

Figure 6 shows the RT Indices for the control, RMA Mix, aged RMA mixes (5-day and 10-day), and the corresponding soybean oil-rejuvenated mixes. The control mixture exhibited the greatest rutting resistance. Conversely, the unaged RMA mixture showed an appreciable decrease in the RT index, indicating that rubber modification enhances the mixture’s elasticity but reduces its shear resistance. LTA notably improved rutting performance: the 5-day- and 10-day-aged RMA mixtures achieved higher RT Index values, demonstrating that oxidative stiffening significantly enhances permanent deformation resistance [29,36]. Rejuvenation with soybean oil resulted in the lowest RT index values among all mixtures tested. The RT Index decreased by 70% for the 5-day aged mixture and by 52% for the 10-day aged mixture after rejuvenation. This confirms that the softening effect of soybean oil drastically reduces the stiffness of aged RMA mixtures, thereby reducing rutting resistance.

### 3.3. Performance Space Diagrams

#### 3.3.1. HWTT vs. DCT Performance Space Diagram

**Figure 7:**
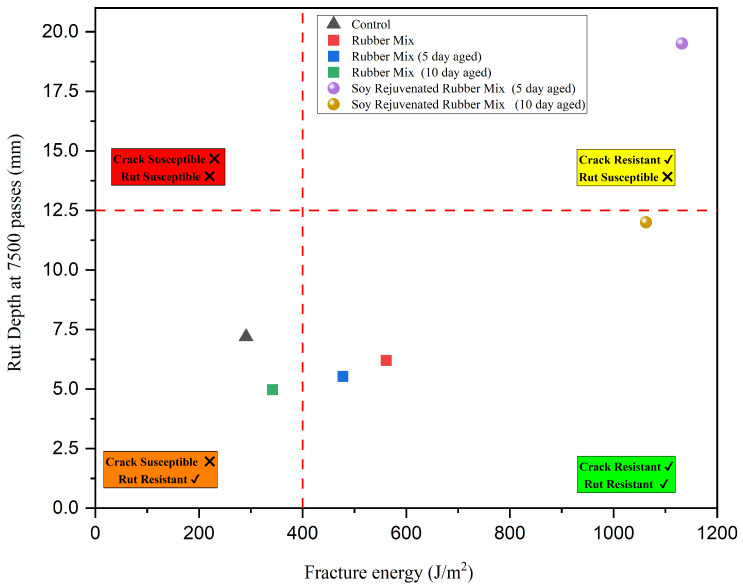
HWTT vs. DCT performance space diagram.

The HWTT vs. DCT performance space diagram shown in Figure 7 summarizes the effects of rubber modification, LTA, and soybean oil rejuvenation observed in this study. The DCT fracture energy of 400 J/m^2^ [8,38] and the HWTT rut depth of 12.5 mm [8,20,39] were selected as the threshold values based on the relevant literature. Briefly, 50% of the mixtures (RMA mix, RMA mix (5-day aged), and soybean oil rejuvenated RMA mix (10-day aged)) provide adequate crack and rutting resistance. The RMA mix lies in the region that meets both crack resistance and fracture energy requirements. This can be attributed to improvements in elastic recovery and energy dissipation when crumb rubber is used to modify asphalt mixtures [2,4,28]. After 5- and 10-day aging at 85 °C, the data shift leftward, indicating lower fracture energy and a downward trend towards lower rut depth, indicating improved rutting performance. The improved rutting can be attributed to hardening of the mixtures due to oxidative aging, which occurs as a result of the loss of light fractions (saturates and aromatics) and the restructuring of asphaltenes. The hardening leads to increased stiffness and reduced ductility, which accounts for the lower fracture energy in the aged rubber mixtures. After the addition of soybean oil to the aged rubber mixtures, the rejuvenated mixtures moved upward and to the right, indicating a major recovery of fracture energy, and simultaneously, the rut depth increased. The recovered fracture energy is due to the soybean oil replenishing the light fractions and enhancing molecular mobility [15,16,40]. However, this softens the binder, leading to increased rutting. The same soybean oil dosage was used for both the 5-day and 10-day soybean-rejuvenated RMA mixtures. This made the 5-day mixture rut-susceptible since it was not as stiffened as the 10-day RMA mixture. A reduced dosage for soybean oil rejuvenation is needed for the 5-day RMA mixture.

#### 3.3.2. IDEAL-RT vs. IDEAL-CT Performance Space Diagram

**Figure 8:**
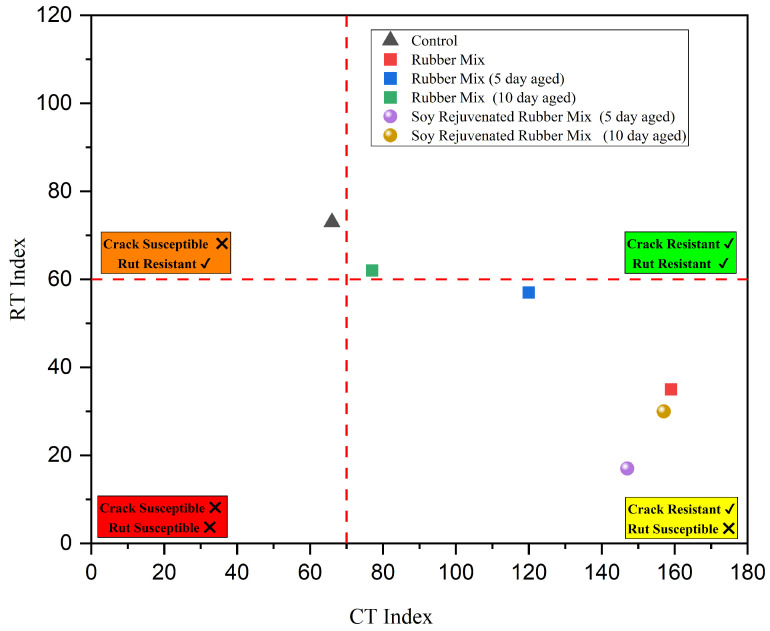
IDEAL-RT vs. IDEAL-CT performance space diagram.

The IDEAL-RT vs. IDEAL-CT performance space diagram shown in Figure 8 summarizes the effects of rubber modification, LTA, and soybean oil rejuvenation observed in this study. Based on the relevant literature, the RT Index of 60 [39,41] and the CT Index of 70 [42] were selected as the threshold values. The 10-day-aged RMA mix meets both cracking and rutting resistance thresholds. The control and the 5-day-aged RMA mix are on the borderline of the RT and CT Index threshold values; as such, they can provide adequate rutting and cracking. It was observed that rejuvenation with soybean oil restores cracking resistance while reducing rut resistance. This can be attributed to the softening of mixtures upon the introduction of light fractions when soybean oil is added [15,16,17,40].

### 3.4. Correlation Analysis of Performance Indicators

The relationships between the four performance indicators (DCT fracture energy, HWTT rut depth at 7500 passes, CT Index, and RT Index) were evaluated using statistical analysis in OriginPro 2025. Both Pearson’s correlation coefficient and Kendall’s rank correlation coefficient were adopted to evaluate the statistical relationships among the variables.

#### 3.4.1. Pearson’s Correlation Analysis

Linear associations between continuous performance metrics were assessed using the Pearson correlation coefficient, which quantifies both the strength and direction of linear relationships on a standardized scale ranging from −1 to +1. Positive values indicate increasing linear dependence, negative values reflect inverse relationships, and values close to zero signify little to no linear correlation [43].

**Figure 9:**
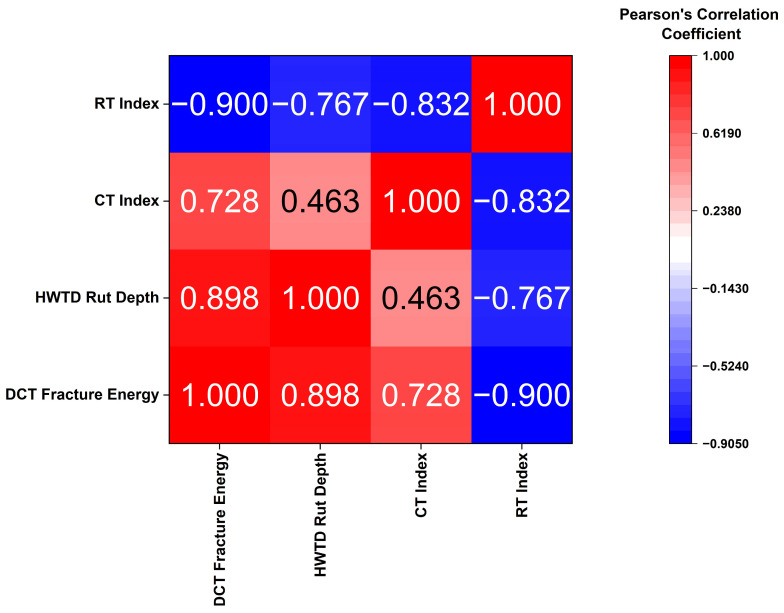
Pearson’s correlation between different performance indicators.

From Pearson’s correlation matrix summarized in Figure 9, a strong positive correlation was found between DCT fracture energy and HWTT rut depth (r = 0.898). Typically, mixtures with higher fracture energy are softer or have lower stiffness. Such mixtures accumulate higher rut depths due to their soft nature. This mirrors the trend from the performance space diagram: when soybean oil is used to rejuvenate aged rubber mixtures, cracking resistance improves, while rutting resistance decreases. Similarly, a strong positive correlation was found between DCT fracture energy and CT Index (r = 0.728). This confirms that mixtures with enhanced low-temperature cracking resistance, as measured by the DCT test, also show improved intermediate cracking resistance for IDEAL-CT testing.

In contrast, strong negative correlations occur between the RT Index and cracking-related indices: CT Index (r = −0.832) and DCT fracture energy (r = −0.900). Additionally, a strong negative correlation was observed between RT-Index and HWTT rut depth (r = −0.767). This confirms the consistency in rutting measurements across different testing modes. Higher rut depths directly correspond to lower RT index, supporting the reliability of using these indicators to evaluate rutting in rejuvenated aged RMA mixtures.

#### 3.4.2. Kendall’s Correlation Analysis

Kendall’s rank correlation coefficient was employed to quantify the ordinal association between paired performance parameters. This nonparametric statistic evaluates the degree of agreement between the rankings of two variables, thereby assessing the strength and direction of their monotonic relationship. Kendall’s rank correlation coefficient (τ) ranges from −1 to +1, with values of greater absolute magnitude indicating stronger ordinal correlation [44].

**Figure 10:**
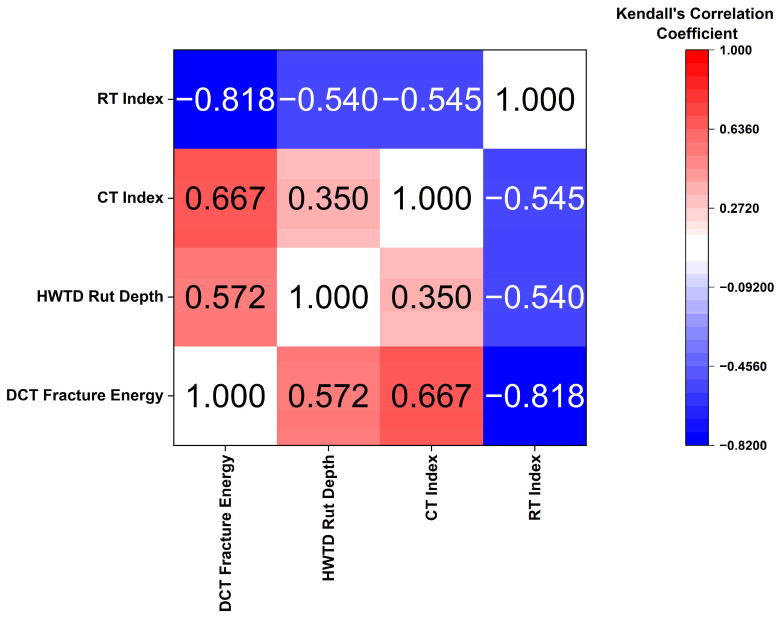
Kendall’s correlation between performance indicators.

From Kendall’s correlation matrix presented in Figure 10, a strong positive correlation was observed between DCT fracture energy and CT index (τ = 0.667). This indicates that mixtures with high low-temperature cracking resistance will also perform well in intermediate cracking. HWTT rut-depth shows a moderately positive correlation with DCT fracture energy (τ = 0.572). This is because softer mixtures, which are crack-resistant, tend to accumulate higher rut depths.

Conversely, strong negative correlations occur between the RT Index and cracking-related indices: DCT fracture energy (τ = −0.818) and CT Index (τ = −0.545). A similar negative association was observed between RT Index and HWTT rut depth (τ = −0.540), consistent with higher rut depths indicating lower rutting resistance across both test methods.

## 4. Conclusions

This study examined the rutting and cracking performance of unaged, long-term-aged, and soybean oil-rejuvenated RMA mixtures. RMA mixtures aged for 5 and 10 days at 85 °C, along with their corresponding soybean-rejuvenated counterparts, were compared with an unaged RMA mixture and a conventional control mixture to assess the combined effects of aging and rejuvenation on RMA mixture performance. Based on the experimental results obtained from the DCT, HWTT, IDEAL-CT, and IDEAL-RT tests, the following conclusions are drawn:

1. Soybean oil rejuvenator effectively restores the cracking resistance of aged RMA mixtures. Based on the DCT and IDEAL-CT results, rejuvenation at 10% of binder weight successfully recovered, and in some cases exceeded, the resistance to cracking of the unaged RMA mixture. This improvement reflects the ability of soybean oil to diffuse into the aged binder and replenish lost light fractions. The resulting increase in flexibility enhances resistance to crack initiation and propagation. Overall, soybean oil provides an efficient restorative pathway for aged RMA systems.
2. Rejuvenation improved the cracking resistance of the aged mixtures but reduced their rutting resistance, demonstrating a clear trade-off in performance. The soybean-treated aged mixtures exhibited higher rut depths and lower RT Index values, confirming that the softening effect introduced by the rejuvenator increased susceptibility to permanent deformation, particularly under water-immersed or elevated-temperature loading conditions.
3. Performance space diagrams further illustrate that an appropriate balance among modification, aging, and rejuvenation can yield mixtures with desirable mechanical behavior. In both the IDEAL-RT vs. IDEAL-CT and HWTT vs. DCT performance spaces, all soybean-rejuvenated mixtures met the cracking performance thresholds, demonstrating the rejuvenator’s strong restorative capability. However, these mixtures did not meet, or only marginally met, the rutting criteria due to the softening effect of soybean oil. This finding suggests that a reduced rejuvenator dosage would likely enable the mixtures to simultaneously satisfy both cracking and rutting requirements, thereby achieving a more balanced overall performance.
4. The correlation analysis confirmed a strong and consistent trade-off between cracking and rutting performance: mixtures with higher fracture energy and CT Index generally showed increased rut susceptibility, while rut-resistant mixtures demonstrated reduced cracking capacity, indicating that balanced performance will require careful optimization of rejuvenator dosage.
5. The use of soybean-oil rejuvenation in long-term-aged RMA mixtures also contributes to climate neutrality by reducing reliance on virgin binder, increasing recycled-material utilization, and extending pavement service life. These effects collectively lower the embodied carbon of asphalt mixtures while supporting circular-economy practices. Thus, bio-rejuvenated RMA offers both performance benefits and measurable contributions to more sustainable, low-carbon pavement systems.

## 5. Recommendations for Future Research

Overall, the results show that soybean-based rejuvenators are effective at restoring the mechanical performance of long-term-aged RMA mixtures. However, the observed trade-off between rutting and cracking performance highlights the importance of carefully controlling rejuvenator dosage to achieve a balanced mixture response suitable for field implementation. To enhance knowledge and support practical implementation of recycled aged RMA mixtures treated with soybean and other bio-based oils, future research should consider the following directions:

1. Investigation of the combinations of soybean and other bio-based oils with polymers, nanomaterials, and anti-stripping agents to concurrently enhance rutting resistance, moisture resistance, and cracking performance.
2. The long-term durability of rejuvenated mixtures should be further investigated. In particular, aging resistance after rejuvenation should be evaluated using extended conditioning protocols to determine whether the softened binders undergo accelerated re-hardening.
3. The dosage of soybean and bio-based oils needs to be optimized across different degrees of oxidative aging to prevent rutting while ensuring adequate cracking recovery.
4. Full-scale field validation is needed. Pilot pavement sections incorporating the rejuvenated aged RMA mixtures should be constructed and monitored to evaluate rutting, cracking, moisture susceptibility, and structural performance under actual traffic and climatic conditions.

## Figures and Tables

**Table 1:** MDOT control mixture gradation used in the study.

Final Mixture Blend Proportion		Aggregate Proportion	
Sieve Size	Percent Passing	Material Type	Percent
1-1/2 inch (37.5 mm)	100%	3/8 WCF	11%
1 inch (25.0 mm)	100%	Dock Screen	14%
3/4 inch (19.0 mm)	100%	SP7 Slag	11%
1/2 inch (12.5 mm)	93.80%	SS 2NS	21%
3/8 inch (9.5 mm)	89.30%	31 A	16%
No. 4 (4.75 mm)	72.90%	5/8 Clear	9%
No. 8 (2.36 mm)	50.30%	RAP	18%
No. 16 (1.18 mm)	36.20%		
No. 30 (0.60 mm)	25.30%		
No. 50 (0.30 mm)	13.90%		
No. 100 (0.15 mm)	7.40%		
No. 200 (0.075 mm)	4.90%		

**Table 2:** RMA mixture gradation used in the study.

Final Blend Proportion		Aggregate Proportion	
Sieve Size	Percent Passing	Material Type	Percent
1-1/2 inch (37.5 mm)	100%	3/8 WCF	19%
1 inch (25.0 mm)	100%	Dock Screen	7%
3/4 inch (19.0 mm)	100%	SP7 Slag	10%
1/2 inch (12.5 mm)	99.70%	SS 2NS	21%
3/8 inch (9.5 mm)	97.50%	31 A	10%
No. 4 (4.75 mm)	79.30%	Trap Sand	13%
No. 8 (2.36 mm)	54.90%	RAP	20%
No. 16 (1.18 mm)	39.30%		
No. 30 (0.60 mm)	27.60%		
No. 50 (0.30 mm)	15.40%		
No. 100 (0.15 mm)	8.20%		
No. 200 (0.075 mm)	5.30%		

## Data Availability

The original contributions presented in this study are included in the article. Further inquiries can be directed to the corresponding authors.
